# Characterization of metabolic responses, genetic variations, and microsatellite instability in ammonia-stressed CHO cells grown in fed-batch cultures

**DOI:** 10.1186/s12896-020-00667-2

**Published:** 2021-01-08

**Authors:** Dylan G. Chitwood, Qinghua Wang, Kathryn Elliott, Aiyana Bullock, Dwon Jordana, Zhigang Li, Cathy Wu, Sarah W. Harcum, Christopher A. Saski

**Affiliations:** 1 Department of Bioengineering, College of Engineering, Computing and Applied Sciences, Clemson University, Clemson, SC 29634 USA; 2 Center for Bioinformatics & Computational Biology, University of Delaware, Newark, DE 19716 USA; 3 Department of Biological Sciences, College of Agriculture, Science & Technology, Delaware State University, Dover, DE 19901 USA; 4 Department of Biological Sciences, Grambling State University, Grambling, LA 71245 USA; 5 Department of Plant and Environmental Sciences, College of Agriculture, Forestry and Life Sciences, Clemson University, Clemson, SC 29634 USA

**Keywords:** CHO, Ammonia, MSI, Genome instability, Biomarker

## Abstract

**Background:**

As bioprocess intensification has increased over the last 30 years, yields from mammalian cell processes have increased from 10’s of milligrams to over 10’s of grams per liter. Most of these gains in productivity can be attributed to increasing cell densities within bioreactors. As such, strategies have been developed to minimize accumulation of metabolic wastes, such as lactate and ammonia. Unfortunately, neither cell growth nor biopharmaceutical production can occur without some waste metabolite accumulation. Inevitably, metabolic waste accumulation leads to decline and termination of the culture. While it is understood that the accumulation of these unwanted compounds imparts a suboptimal culture environment, little is known about the genotoxic properties of these compounds that may lead to global genome instability. In this study, we examined the effects of high and moderate extracellular ammonia on the physiology and genomic integrity of Chinese hamster ovary (CHO) cells.

**Results:**

Through whole genome sequencing, we discovered 2394 variant sites within functional genes comprised of both single nucleotide polymorphisms and insertion/deletion mutations as a result of ammonia stress with high or moderate impact on functional genes. Furthermore, several of these de novo mutations were found in genes whose functions are to maintain genome stability, such as *Tp53, Tnfsf11, Brca1,* as well as *Nfkb1.* Furthermore, we characterized microsatellite content of the cultures using the CriGri-PICR Chinese hamster genome assembly and discovered an abundance of microsatellite loci that are not replicated faithfully in the ammonia-stressed cultures. Unfaithful replication of these loci is a signature of microsatellite instability. With rigorous filtering, we found 124 candidate microsatellite loci that may be suitable for further investigation to determine whether these loci may be reliable biomarkers to predict genome instability in CHO cultures.

**Conclusion:**

This study advances our knowledge with regards to the effects of ammonia accumulation on CHO cell culture performance by identifying ammonia-sensitive genes linked to genome stability and lays the foundation for the development of a new diagnostic tool for assessing genome stability.

**Supplementary Information:**

The online version contains supplementary material available at 10.1186/s12896-020-00667-2.

## Background

Biopharmaceutical manufacturing represents nearly 2% of the total US GDP [1] which makes it an important driver of the US economy. Biopharmaceuticals include monoclonal antibodies, recombinant proteins, and assemblies of proteins produced by biological means. Commercial products are used as blood factors, thrombolytic agents, therapeutics, growth factors, interferons, and vaccines [2, 3]. The most common mammalian cell line used is the Chinese hamster ovary (CHO) cell line, due to its ability to produce biopharmaceutical molecules with post-translational modifications required in humans [4]. However, it is well understood that recombinant CHO cell lines are susceptible to genome instability that is often observed after approximately 70 generations [5–8]. Previous studies have characterized genomic variants across various CHO cell lines that may be a contributing factor to genome instability [9–11]. An unstable genome can result in reduced productivity in continuous cultures and fed-batch systems [12, 13]. A common occurrence in both continuous cultures and fed-batch systems is the accumulation of metabolic waste products, such as ammonia and lactate. The role these waste products play in cellular processes, such as glycosylation, metabolism, and productivity have been characterized [14–17]; however, the effects of these waste products on genome stability have not been directly assessed.

Microsatellite instability (MSI) is described as genetic hypermutability at microsatellite loci where a high frequency of insertion or deletion (indel) mutations accumulate in daughter cells during cell division [18, 19]. MSI results from improperly functioning mismatch repair (MMR) pathways which are key to maintaining genome stability [20]. Rather than correcting DNA mismatch errors that occur spontaneously during DNA replication, cells with impaired MMR systems accumulate these errors over the course of subsequent propagation. The prevalence of these errors allow for MSI loci to be utilized as stable genetic biomarkers that are capable of diagnosing many human cancers [21, 22]. Studies have shown that approximately 15% of human patients with colorectal cancer [20, 23], 20% of patients with stomach cancer [24], and 30% of patients with endometrial cancer [25] could attribute their disease to genome instability that can be diagnosed with MSI biomarkers. The clinical uptake of MSI-based diagnostics, such as the Bethesda Panel, demonstrates the reliability and clinical utility of MSI loci as biomarkers [26].

In this study, we investigated the effects of exogenous ammonia exposure on genome stability during fed-batch cultures of CHO cells. Specifically, the accumulation of DNA mutations in cells exposed to elevated ammonia were compared to cultures grown under standard fed-batch conditions. Ammonia was added to duplicate parallel cultures at 10 mM and 30 mM final concentrations at 12 h of culture time to establish mild and high ammonia stresses respectively. After 72 h of elevated ammonia exposure, samples were taken for whole genome sequencing (WGS). These sequences were then analyzed for MSI, single nucleotide polymorphisms (SNPs), and insertion/deletion (indel) variations. The SNPs and indels were mapped to the Chinese hamster genome and assessed for functional impact in both coding and regulatory genetic regions. Microsatellite regions were analyzed to identify loci with dose-dependent indel mutations that could be used as potential biomarkers.

## Materials and methods

### Culture conditions

A recombinant CHO-K1 Clone A11 from the Vaccine Research Center at the National Institutes of Health (NIH), which expresses the anti-HIV antibody VRC01 (IgG_1_) was used. The inoculum train was expanded in 250 mL shake flasks with 70 mL ActiPro media (GE Healthcare) that were maintained at 5% CO_2_ and 37 °C. The bioreactors were ambr® 250 bioreactors (Sartorius Stedim, Göttingen, Germany) with two pitched blade impellers and an open pipe sparger (vessel part number: 001-5G25). The bioreactors were inoculated at a target cell density of 0.4 × 10^6^ cells/mL in ActiPro batch media and fed daily beginning on Day 3 (3% (v/v) Boost 7a and 0.3% (v/v) Boost 7b (GE Healthcare)). Duplicate cultures were ammonia-stressed at 12-h post inoculation with 0 mM, 10 mM, or 30 mM NH_4_Cl. The 0 mM and 10 mM cultures used saline to normalize the volume of the 0 mM and 10 mM cultures to the 30 mM cultures. Dissolved oxygen was controlled at 50% of air saturation using PID control that increased the O_2_ mixture in the gas sparge to 100%, then the stir speed from 300 to 600 rpm. Antifoam (10% solution in media; SH30897.41 – GE Healthcare) was added as needed to control foaming. All gases were supplied through the open pipe sparger; an overlay was not used. The pH was controlled via sparging CO_2_ and air, and base pump (1 M NaOH). The pH setpoint was 7.0 with a 0.2 deadband. Temperature was controlled at 37 °C. Samples for WGS and MSI analysis were harvested at 84 h culture time (72 h post-stress) and centrifuged at approximately 2000 x g for 15 min at 4 °C. The supernatant was removed, and the pellet was stored at − 80 °C.

### DNA extraction, whole genome sequencing, and microsatellite variant discovery

Pellets of approximately 0.5 × 10^6^ cells were pre-washed with 1X phosphate buffered saline (PBS) prior to extraction. Total genomic DNA (gDNA) was purified from 2 replicate samples per condition with the DNAeasy Blood and Tissue Kit (Qiagen), following the manufacturer’s recommended procedures and combined prior to sequencing. Whole genome shotgun sequencing was performed on an Illumina NovaSeq (2 × 150 paired end) through a third- party vendor to approximately 30x genome coverage. Raw sequence data was assessed for quality with the FASTQC software (https://www.bioinformatics.babraham.ac.uk/projects/fastqc/). Raw sequence data was preprocessed to remove low quality bases and adapter sequences with the Trimmomatic software v.0.38 [27]. Preprocessed reads were aligned to the CriGri-PICR version assembly of the Chinese hamster genome (*Cricetulus griseus*) (RefSeq assembly accession: GCF_003668045.1) with the Bowtie2 v.2.3.4.1 short read aligner [28]. Alignments were coordinate sorted and indexed with SamTools v1.3.1 [29]. SNPs and indels were determined with the HaploTypeCaller Walker from the Genome Analysis Toolkit (GATK v.4.0) [30]. Genetic variants in the resulting VCF file (SNP and INDEL) were hard filtered according to the following criteria: read depth (DP 6) and mapping quality (MQ 30). Variant sites were further filtered to remove variant loci that were common between the sample groups but differed from the CHO PICR reference assembly. Finally, variant sites were kept in the final VCF file only if one or both of the treatment samples differed from the control. Functional SNPs were characterized with the SnpEff software, v4.3 [31]. Genome-wide microsatellite loci were determined against the PICR CH assembly with MISA, a microsatellite finder software [32]. Microsatellite loci were intersected with indel coordinates using BedTools Intersect command 2.27.1 [33] to identify indel variants associated with microsatellites.

### Text/data mining and functional enrichment analysis

The query “genomic instability [MeSH Terms] “was used to search PubMed to retrieve the abstracts with PMIDs (14,968 PMIDs). The PubTator [34] tool was used to collect genes annotated in these abstracts with Entrez Gene IDs (ftp://ftp.ncbi.nlm.nih.gov/pub/lu/PubTator/gene2pubtator.gz released 2/14/2020). Among the 5073 retrieved genes, 3131 genes were human (*Homo sapiens*), 882 mouse (*Mus musculus*), 435 yeast (*Saccharomyces cerevisiae*) representing the three top species with the largest number of genes mapped in those PubMed abstracts. The other top mapped species include seven vertebrates: rat, chicken, zebrafish, frog, Chinese hamster, dog and pig as well as three non-vertebrates: fly, *Arabidopsis*, and worm. The ortholog pairs between human and the eight other above vertebrates were mapped with NCBI ortholog assignment (ftp://ftp.ncbi.nlm.nih.gov/gene/DATA/gene_orthologs.gz released 07/20/2020), whereas those between human and the four non-vertebrate species were mapped with OMAbrowser (https://omabrowser.org/oma/genomePW/). Altogether, 2897 human genes linked to “genomic instability” were matched with corresponding Chinese hamster orthologs. For the SNP list with high and moderate impact mutations by SnpEff, 273 Chinese hamster genes were mapped with their corresponding human orthologs that had been linked to “genomic instability”. ClusterProfiler [35, 36] was used to obtain the enriched KEGG pathways and GO annotations for the given gene lists.

### Identification of candidate MSI loci

Candidate MSI loci were determined with a filtering strategy that leverages several criteria as follows: First, each novel indel-variable genomic locus was assigned a mutation score which is a proportion of the number of variant reads (allelic depth) by the total depth of reads for each site extracted from the .vcf file. Second, the mutation scores of the control cultures were subtracted from the mutation scores of the treated loci in order to generate a mutation score relative to the control. This allowed for the removal of loci that did not exhibit dose-dependent responses to the exogenous ammonia. Concurrently, loci with nonpositive relative mutation scores were also removed. The remaining loci were then intersected with the genome-wide microsatellite coordinates determined by MISA with the Intersect command of BedTools v2.27.1 [33] to identify loci within known microsatellites. The final ranked set of candidate MSI loci contain sites where control samples have fewer to no variant reads in comparison to the treated samples.

## Results

### Growth and metabolite profiles

**Fig. 1:**
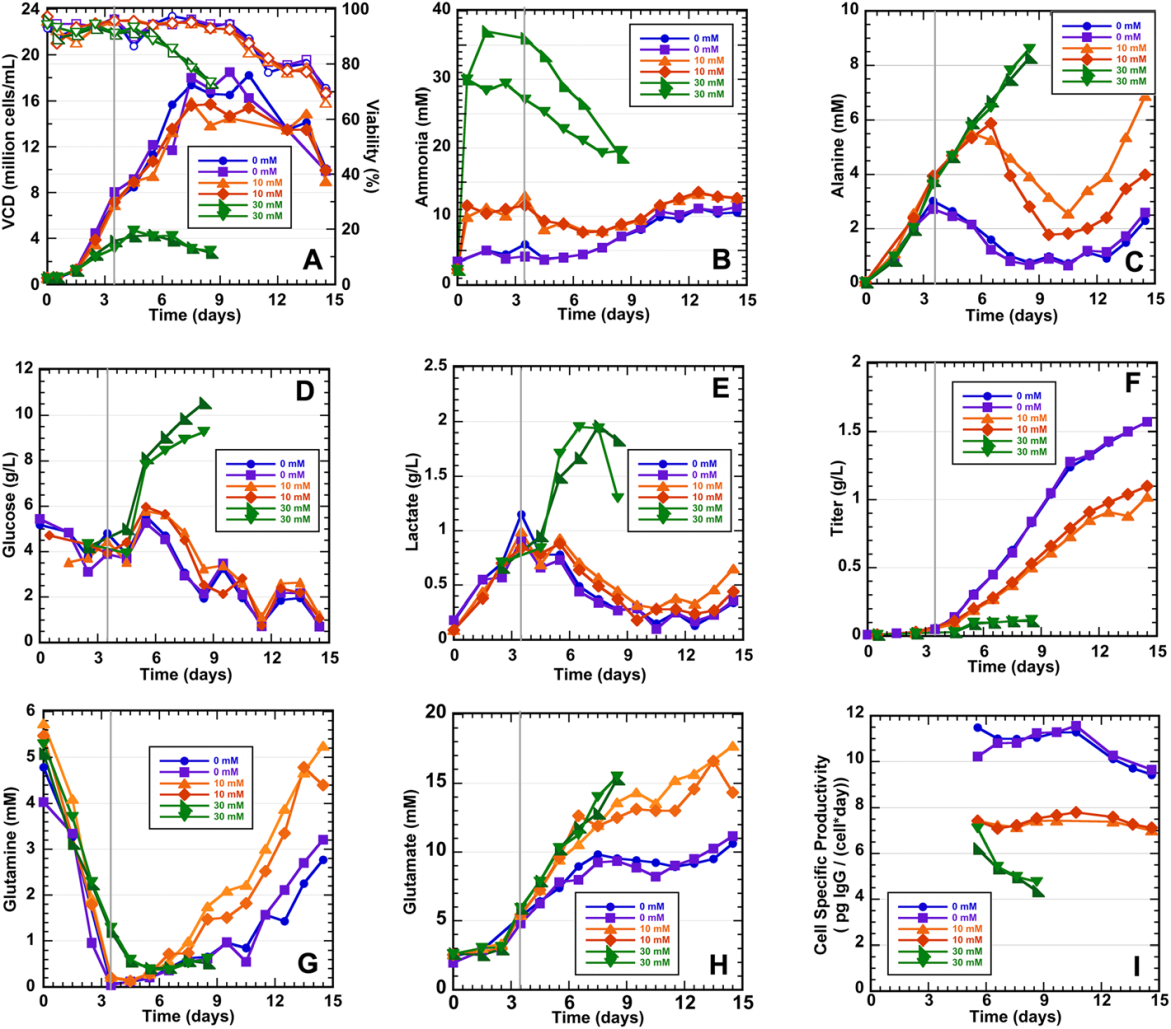
Cell growth, ammonia, titer and metabolic profiles for CHO K-1 VRC01 cells cultured in duplicate in the ambr® 250 bioreactor. The ammonia stresses (10 mM and 30 mM) were added at 12 h. Samples for genomic analysis were harvested at 84 h (3.5 days) as shown by the solid grey line. **a** Viable cell density (VCD) and viability (filled and hollow symbols, respectively), **b** ammonia, **c** alanine, **d** glucose, **e** lactate, **f** titer of recombinant monoclonal antibody, **g** glutamine, **h** glutamate, and **i** Cell specific productivity (qp). Due to low levels of the recombinant protein in culture prior to day 5, the qp value is not shown until a significant titer has been reached. In industry, it is common to only measure titers starting at day 7. Control - 0 mM (blue and purple lines); 10 mM (orange and red lines); 30 mM (green and dark green lines)

Recombinant CHO cells expressing the monoclonal antibody VRC01 were cultured in tightly controlled ambr® 250 bioreactors for 12 h prior to the addition of ammonia to stress the cultures. Up to 1.5 days post-inoculation, there were no observable differences in the viable cell densities (VCD); however, at 2.5 days, the 30 mM ammonia-stressed cultures had substantially lower VCDs compared to the control and 10 mM stressed cultures (Fig. 1a). The 10 mM ammonia-stressed cultures had similar VCDs to the control cultures until Day 7; yet cell viabilities were similar to the control cultures for the entire culture durations. In contrast, the 30 mM ammonia-stressed cultures reached peak VCDs on Day 4 and gradually declined until the cultures were harvested on Day 8.5 due to low viability (< 70%); a cell viability below 70% is a standard harvesting threshold. Samples for genome sequencing were taken 84 h post inoculation (Day 3.5), i.e., 72 h post-stress. At the time of harvest of genome sequencing samples, the viability for all samples was greater than 90% (Fig. 1a). The mildly stressed (10 mM) cultures had no significant change in the ammonia levels between 12 and 84-h, while the high stress (30 mM) cultures had a gradual decline in the ammonia concentration until harvested (Fig. 1b). The glucose and lactate profiles (Fig. 1d, e) confirm that the control and 10 mM ammonia-stressed cultures were paired closely throughout the entire cultures, although the 10 mM stressed culture had slightly lower VCD beginning on Day 6. In contrast, the 30 mM ammonia-stressed cultures began to accumulate glucose and lactate after Day 5, most likely due to the set feeding protocol based on culture volume, and the significantly lower cell growth (Fig. 1a). It is well-known that excessive glucose inevitably leads to lactate accumulation [15], which was observed for the 30 mM stressed cultures. Amino acid profiles were also obtained for these cultures [37]. The amino acid profile that showed the greatest differences between the control and 10 mM cultures was alanine; both the 10 mM and 30 mM ammonia-stressed culture alanine profiles were very similar through Day 6, while the control cultures had profiles that represented a higher consumption rate, as alanine was fed starting on Day 3 (Fig. 1c). The glutamine profiles for the control and 10 mM cultures were similar up to Day 7, until the 10 mM cultures began to accumulate glutamine (Fig. 1g). The glutamine accumulation can be attributed to the feeding of glutamate (Fig. 1h), which when in excess can be aminated to form glutamine [38]. The 30 mM cultures were terminated prior to any significant differences in the glutamine accumulation being observed between the control and 30 mM cultures. Therefore, the glutamate feeding, based on volume, caused glutamate to accumulate due to lower VCD relative to the control cultures for the 10 mM and 30 mM cultures. This in turn impacted the glutamine profile. A global measure of cell health is the overall protein production and cell-specific productivity. The monoclonal antibody titer at the end of the cultures was about 50% lower for the 10 mM cultures compared to the control cultures, whereas the 30 mM cultures had negligible protein productivity in (Fig. 1f). Furthermore, cell-specific productivity (picograms of IgG per cell per day) was found to be substantially higher in the control cultures when compared to the 10 mM sample. It should also be noted that the control and 10 mM cultures had a relatively stable production rate, whereas the 30 mM cultures declined (Fig. 1i). Overall, the samples for the genome sequencing analysis were taken at culture times when there were no substantial VCD, viability, or metabolic differences between the control and 10 mM ammonia-stressed cultures; however, the VCD was significantly lower for the 30 mM ammonia-stressed cultures (Fig. 1).

### Whole genome shotgun sequencing and variant discovery in stressed conditions

**Fig. 2:**
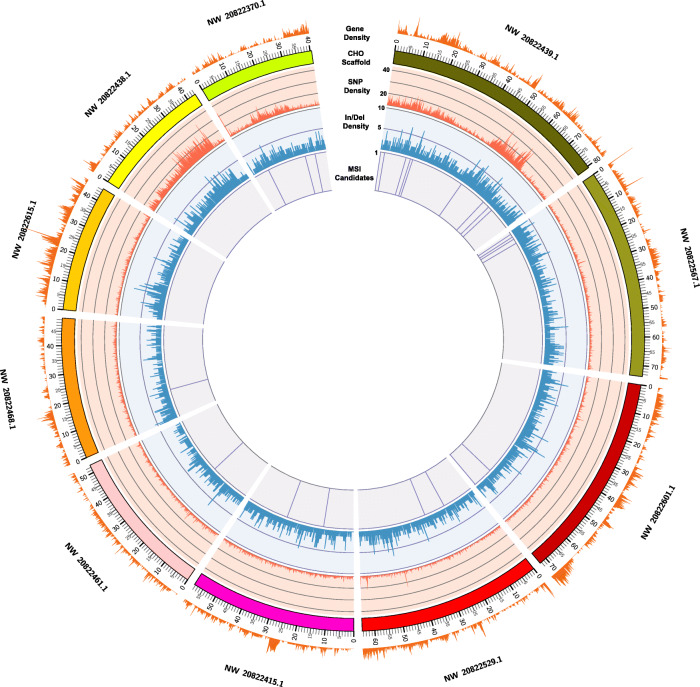
Genome coverage map of genetic variants and MSI loci in the 10 longest CHO scaffolds. A circos plot of the 10 longest CHO genome scaffolds (parsed into 100 kb windows that represents approximately ~ 20% of the CHO genome) that depicts distribution of genetic variants and MSI loci. The innermost track (light purple) depicts candidate MSI loci (23 out of the 124); Indel density and distribution is depicted in light blue; SNP density and distribution is in light red; and gene density is plotted outside of the CHO ideograms in light orange. Ideogram ticks are scaled in megabases

Whole genome shotgun sequences were collected for the control and treated samples to an approximate depth of 30X coverage to assess the genomic impact of exogenous ammonia exposure. A total of 389,694 variant sites were identified across both stress levels that were composed of 310,597 SNPs and 79,097 indels, (Supplemental Tables S1 and S2 respectively). Of the 389,694 variant sites, a total of 135,913 variant sites reside in protein coding genes (Supplemental Table S3). The variant sites were seemingly randomly distributed in both intergenic and genic positions across the genome. A distribution and density map of variant positions relative to annotated coding genes is depicted in Fig. 2. These variants were further filtered to remove sites annotated as a modifier or low impact variant (e.g. synonymous mutations) predicted by SnpEff. This led to the discovery of 2394 variants within protein coding genes with significant impact variations (high/moderate impact predicted by SnpEff) due to ammonia stress (Supplemental Table S4).

### Functional impact of ammonia-induced variants in genome stability genes

**Fig. 3:**
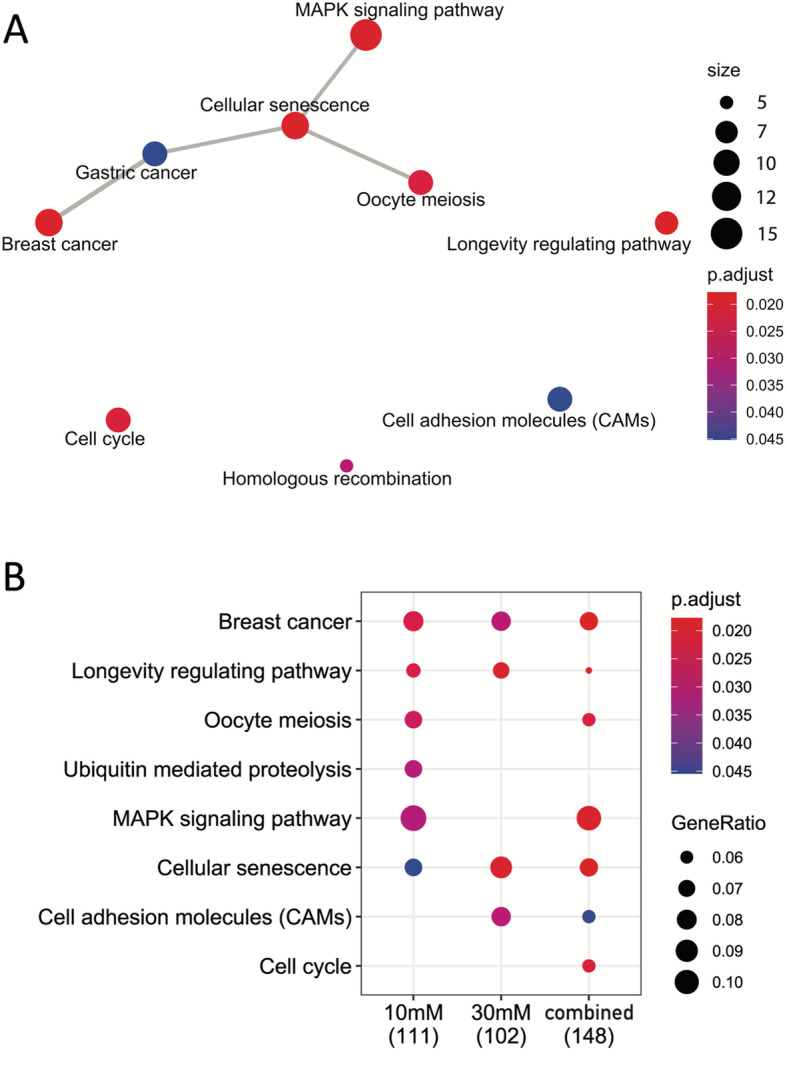
KEGG enrichment results from over representation analysis of ammonia-sensitive genes linked to genomic instability. **a** Network plot of most significant enriched KEGG pathways. Enrichment scores (i.e., adjusted *p* values) and gene counts (the number of genes enriched in a KEGG term) are depicted by dot color and size. **b** Comparison of enrichment results of KEGG pathways for genes identified with significant variants in the 10 mM Ammonia-stressed culture (111 genes), in the 30 mM culture (102 genes), and in the union of 10 mM and 30 mM cultures (148 genes). Enrichment scores (i.e., adjusted p values) and gene ratios (the percentage of total genes in the given KEGG term) are depicted by dot color and size. The plots are made with clusterProfiler

**Fig. 4:**
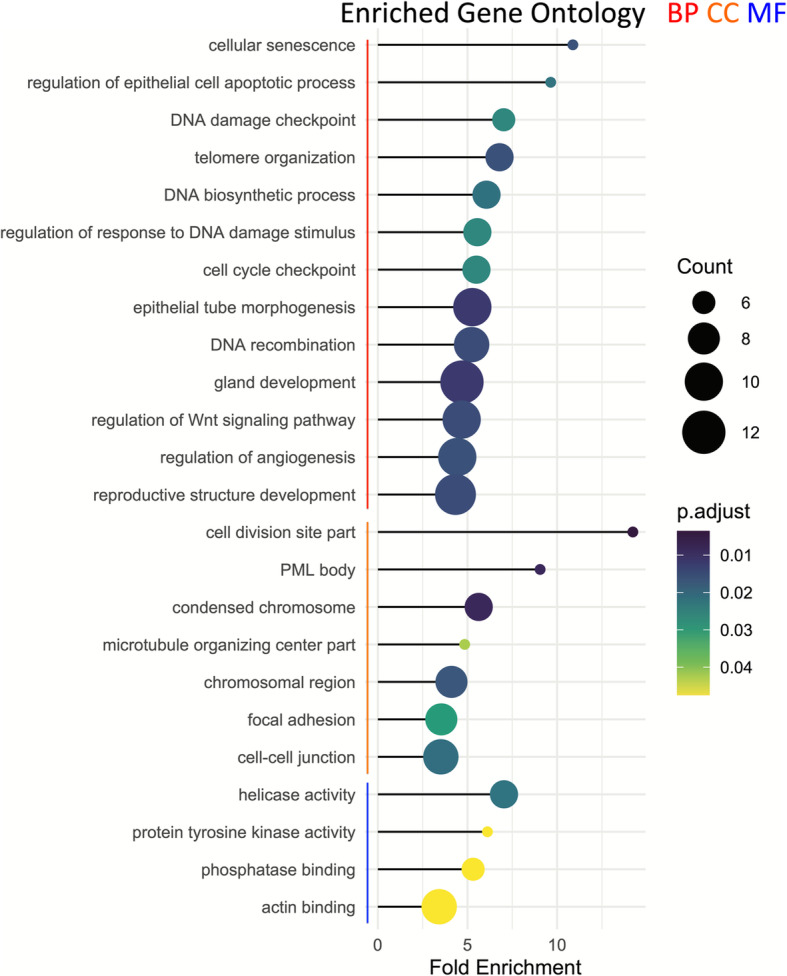
Gene Ontology (GO) enrichment results from over representation analysis of ammonia-sensitive genes linked to genomic instability. Terms in the three GO categories are grouped with color bars: biological process (BP, red), cellular component (CC, orange), and molecular function (MF, blue). Fold enrichment is shown in x-axis. Enrichment scores (i.e., adjusted p values) and gene counts (the number of genes enriched in a GO term) are depicted by dot color and size. The plot is made with clusterProfiler

The above described 2394 variants were assigned to 1843 Chinese hamster protein-coding genes with certain functional impact. Through mapping of human orthologs for those Chinese hamster genes, we found 273 genes that are linked to genome instability terms via text mining (Supplemental Table S5). Figure 3a shows the map for KEGG enrichment result of over-representation test of the 273 genes. The five most significant KEGG pathways include breast cancer, cellular senescence, longevity regulating pathway, MAPK signaling pathway, and cell cycle. It is critical to note that the KEGG enrichment analysis (Fig. 3a) combines all variants found in the 10 mM and 30 mM stress cultures, whereas gene lists for variants exclusively detected in 10 mM or 30 mM stress samples generated no enrichment of KEGG pathways. Fig. 3b shows the KEGG comparison between three gene lists: one for all variants in 10 mM sample, another for 30 mM sample, and one for variants from the combined list (i.e., the above mentioned 273 genes). Breast cancer, cellular senescence, longevity regulating pathway are the three KEGG pathways common to the three gene lists (Fig. 3b). These corresponding genes are listed in Table 1. Figure 4 summarizes the significant GO terms enriched among the genes for variants existing at both stress levels. Notable GO biological process terms in Fig. 4 include DNA recombination, cell cycle checkpoint, regulation of response to DNA damage stimulus, telomere organization, and DNA damage checkpoint. Additionally, notable functions of genes include double-strand break repair (*Brca1*), mismatch repair (*Mlh3*), and centromere generation (*Cenpc*) (Supplemental Table S5). More detailed enrichment analysis on KEGG and GO of variant genes can be found in Supplemental Tables S6, S7, S8 and S9.

**Table 1:** A summary of select KEGG enrichment genes discovered in ammonia-stressed cultures that can be linked to genome instability in humans via text mining

Gene Name (Human)	Entrez Gene ID (Human)	Entrez Gene ID (Chinese hamster)
Tumor necrosis factor superfamily member 11 (TNFSF11)	8600	100,768,715
Peroxisome proliferator activated receptor gamma (PPARG)	5468	100,689,245
Interleukin 1 alpha (IL1A)	3552	100,769,260
Wnt family member 1 (WNT1)	7471	100,766,046
Protein phosphatase 1 catalytic subunit alpha (PPP1CA)	5499	100,760,810
Transforming growth factor beta receptor 1 (TGFBR1)	7046	100,772,727
E2F transcription factor 4 (E2F4)	1874	100,765,561
Frizzled class receptor 2 (FZD2)	2535	100,763,109
LDL receptor related protein 6 (LRP6)	4040	100,772,150
Tuberous sclerosis 2 (TSC2)	7249	100,755,849
lin-9 DREAM MuvB core complex component (LIN9)	286,826	100,774,401
BRCA1, DNA repair associated (BRCA1)	672	100,770,724
Mitogen-activated protein kinase kinase 1 (MAP 2 K1)	5604	100,689,403
Protein kinase AMP-activated non-catalytic subunit gamma 3 (PRKAG3)	53,632	100,770,459
Activating transcription factor 2 (ATF2)	1386	100,754,663
RB1 inducible coiled-coil 1 (RB1CC1)	9821	100,763,340
Progesterone receptor (PGR)	5241	100,757,656
fms related tyrosine kinase 4 (FLT4)	2324	100,766,609
Klotho (KL)	9365	100,758,189
Notch 1 (NOTCH1)	4851	100,761,880
Beta-transducin repeat containing E3 ubiquitin protein ligase (BTRC)	8945	100,750,426
Nuclear factor kappa B subunit 1 (NFKB1)	4790	100,770,607
Tumor protein p53 (TP53)	7157	100,682,525
Cyclin E1 (CCNE1)	898	100,753,358
Notch 2 (NOTCH2)	4853	100,771,788

### Microsatellite and candidate MSI loci

**Fig. 5:**
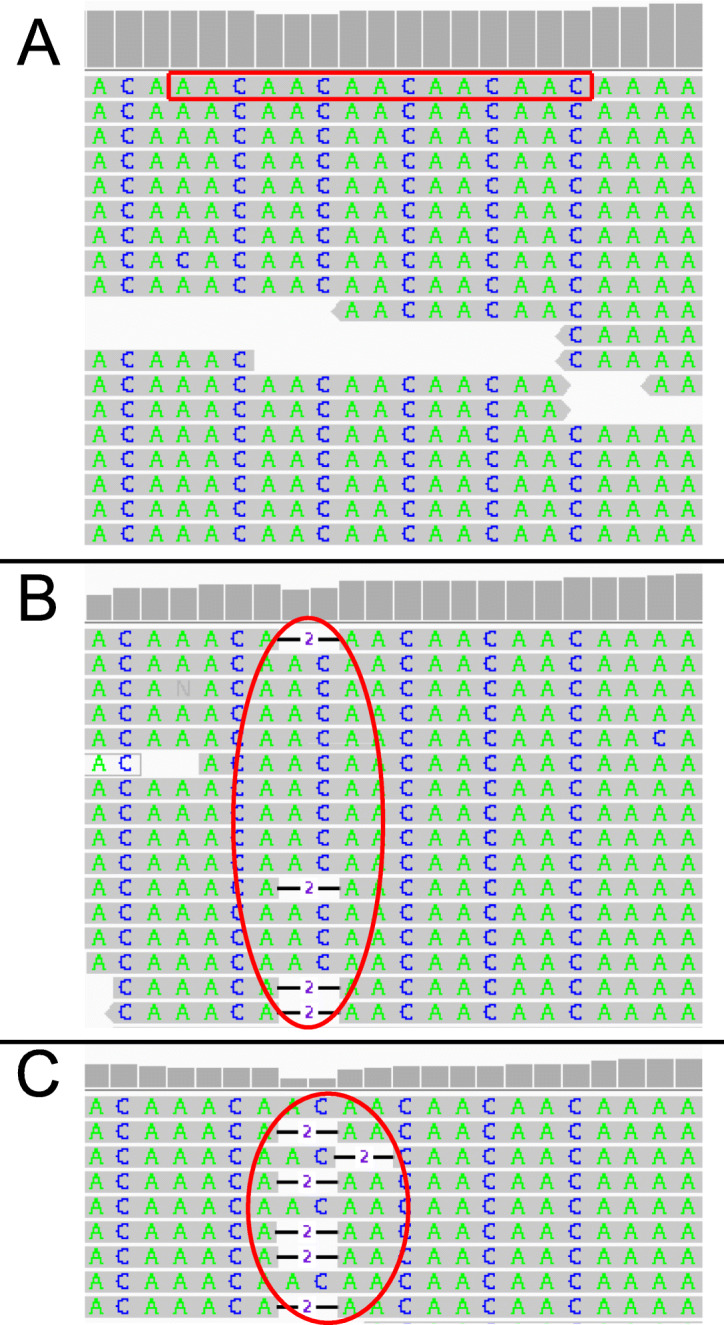
An Integrated Genome Viewer (IGV) image of a microsatellite located on scaffold NW_020822544.1 at position 4,160,116. **a** control, **b** 10 mM, and **c** 30 mM cultures. This microsatellite contains five repeats of an AAC motif. They grey lines above each nucleotide are indicative of the total read depth at that location; note that it is significantly lower in regions where deletions were detected

A whole-genome scan for microsatellites discovered a total of 409,628 loci, with motifs that included di-, tri-, and tetranucleotide repeats (Supplemental Table S10). As expected, the microsatellites composed of dinucleotide repeats were the most prevalent with a total of 287,124. Trinucleotide and tetranucleotide motifs were less abundant with 46,602 and 75,902 occurrences, respectively. An analysis of genome-wide indels in ammonia treated and control samples revealed 1022 microsatellites that were lengthened or shortened due to the ammonia stress (Supplemental Table S11). An example microsatellite locus with desirable length variation resulting from elevated ammonia is shown in Fig. 5. In this example, there is a higher abundance of mapped reads with deletions for the 30 mM ammonia stressed cultures, suggesting a dose-dependent response. Furthermore, we developed a custom mutation score and stringent filtering criteria (see Methods) to identify a candidate set of 124 MSI loci where stable mutations were present in both ammonia-stressed cultures, but were not present in the control cultures. These 124 MSI loci can be used as a foundation for future research as diagnostics for genome instability (Fig. 2). It is important to note that because the mutation score was calculated using the allelic depth; loci with more reads are statistically more significant than those with fewer reads. With this in mind, the 124 candidate MSI loci may not be all inclusive of the optimal loci due to the variation in mapped read depth across the genome. The remaining loci after each filter step is summarized in Table 2. A full list of loci in each step can be found in Supplemental Tables S12, S13, S14 and S15, while the location of all candidate loci are summarized in Table 3.

**Table 2:** Numerical representation of filter progression

Filter Criteria	Remaining Loci
Genome-wide INDELs	79,097
30 mM Relative Mutation Score > 10 mM Relative Mutation Score	35,437
Loci with positive Relative Mutation Score	16,678
Loci confirmed to be microsatellites	187
Loci with genotypes that vary from the control	124

**Table 3:** Location and composition of all candidate microsatellites. More detailed information on candidate loci can be found in Supplemental Table S15

			Mutation Scores (%)		
Scaffold	Position	Motif	10 mM	30 mM	Control
NW_020822370.1	34,498,325	(GA)31	85.71	94.44	83.33
NW_020822370.1	29,116,483	(AG)22	14.29	62.5	0
NW_020822370.1	8,110,802	(GT)33	14.29	20	7.14
NW_020822373.1	4,520,712	(TC)27	10.34	12.4	4.59
NW_020822375.1	17,984,519	(GT)23	90	100	77.78
NW_020822375.1	24,391,224	(CA)8	41.67	70	0
NW_020822376.1	2,957,094	(CA)7	13.04	21.05	8.33
NW_020822382.1	3,087,333	(CCTC)5	14.29	21.43	0
NW_020822403.1	19,387,296	(AC)25	28.57	50	4.76
NW_020822403.1	15,933,029	(AC)26	25	46.15	0
NW_020822406.1	3,701,096	(GT)23	14.29	42.86	9.09
NW_020822407.1	7,711,795	(AAAC)6	16.67	26.67	0
NW_020822409.1	5,651,224	(AAT)12	14.29	44.44	0
NW_020822410.1	13,888,699	(GT)28	83.33	90.32	63.64
NW_020822410.1	10,858,329	(AC)19	94.12	96.15	75
NW_020822412.1	3,775,426	(TG)26	21.43	28.57	0
NW_020822415.1	36,220,614	(TC)31	91.67	92.31	57.14
NW_020822415.1	16,052,939	(ACA)5	19.23	23.33	5.26
NW_020822421.1	5,779,917	(TC)29	91.3	100	80
NW_020822426.1	5,559,446	(GA)31	93.75	100	90.91
NW_020822426.1	3,827,444	(TCT)30	71.43	72.73	55.56
NW_020822426.1	1,539,468	(GT)22	27.27	28.57	0
NW_020822428.1	1,367,872	(AC)26	93.75	100	85.71
NW_020822428.1	8,085,398	(TCCT)11	16.67	27.27	0
NW_020822434.1	512,274	(GT)28	18.18	44.44	0
NW_020822436.1	3,837,117	(AC)16	90.32	93.33	38
NW_020822439.1	68,421,902	(AC)29	91.67	100	83.33
NW_020822439.1	47,065,858	(TC)35	84.62	94.74	80
NW_020822439.1	62,174,741	(GT)23	22.22	35.48	11.11
NW_020822439.1	14,184,660	(AAAC)7	12	50	0
NW_020822439.1	66,094,811	(AC)24	25	28.57	0
NW_020822439.1	16,584,719	(GT)8	20	36.36	7.14
NW_020822439.1	2,390,672	(TG)62	12.5	33.33	0
NW_020822439.1	243,530	(CA)25	25	50	0
NW_020822439.1	17,884,454	(AGAT)11	13.33	20	7.14
NW_020822440.1	3,560,698	(GA)28	88.89	90	83.33
NW_020822443.1	2,115,154	(AC)27	27.27	43.75	9.09
NW_020822446.1	382,267	(CA)32	66.67	75	33
NW_020822450.1	9,390,312	(AC)22	16.67	44.44	11.11
NW_020822452.1	9,210,374	(CT)31	90	93.33	81.82
NW_020822458.1	15,628,677	(AG)27	91.67	100	73.33
NW_020822461.1	21,799,265	(TCTT)16	16.67	25	0
NW_020822464.1	1,598,541	(AC)26	84.62	100	83.33
NW_020822464.1	6,242,934	(TG)26	27.78	36	0
NW_020822465.1	1,645,147	(TG)9	39.29	54.55	5.88
NW_020822468.1	15,157,395	(GT)35	87.5	100	66.67
NW_020822469.1	3,552,281	(GATG)8	31.25	54.55	0
NW_020822484.1	1,703,831	(TG)7	95.65	100	77.27
NW_020822486.1	16,687,223	(GT)23	21.43	22.22	0
NW_020822487.1	21,884,245	(CT)34	89.66	96.43	80
NW_020822487.1	17,786,455	(TC)30	91.67	100	87.5
NW_020822487.1	25,648,949	(AC)15	25	37.5	10
NW_020822487.1	8,954,697	(CA)29	10	18.18	0
NW_020822488.1	1,550,207	(TG)26	85.71	100	75
NW_020822499.1	2,670,448	(TG)31	95.65	100	84
NW_020822499.1	21,810,339	(CT)20	35.71	56.25	0
NW_020822499.1	16,894,388	(TTA)5	11.9	13.73	6.67
NW_020822499.1	10,821,740	(TTGT)8	14.29	38.46	7.14
NW_020822501.1	11,357,080	(GT)29	91.67	100	85.71
NW_020822501.1	14,044,688	(TTG)7	14.29	24.32	3.33
NW_020822501.1	14,071,142	(TTA)12	20	28.57	5.56
NW_020822501.1	17,408,028	(TG)30	55.56	57.14	7.14
NW_020822503.1	10,248,411	(CTTT)14	22.22	25	0
NW_020822503.1	4,513,778	(GT)14	10.53	20	0
NW_020822503.1	17,353,421	(GT)6	22.22	25	9.09
NW_020822504.1	9,433,369	(TC)30	95	100	88.89
NW_020822504.1	13,319,850	(TGTA)5	12.5	15.56	5.88
NW_020822505.1	16,324,767	(CA)24	93.75	100	77.78
NW_020822505.1	16,510,213	(AC)30	85.71	87.5	77.78
NW_020822505.1	10,642,584	(TG)27	27.27	37.5	0
NW_020822508.1	1,001,369	(AC)22	90.91	100	71.43
NW_020822508.1	2,728,629	(TATC)10	16.67	27.78	0
NW_020822508.1	15,865,728	(GAAG)13	7.69	31.25	0
NW_020822508.1	15,865,731	(GAAG)13	7.14	26.32	0
NW_020822511.1	9,568,885	(TG)29	92.31	100	83.33
NW_020822512.1	6,724,926	(TG)30	87.5	93.33	85.71
NW_020822519.1	6,274,345	(AC)25	91.67	100	84.62
NW_020822519.1	11,516,391	(AG)36	15.79	37.5	0
NW_020822520.1	2,511,201	(AC)24	20	25	0
NW_020822526.1	18,794,354	(AC)26	91.67	100	83.33
NW_020822526.1	25,646,840	(CA)7	90	100	77.78
NW_020822526.1	24,206,438	(GT)30	87.5	90	70
NW_020822526.1	16,873,752	(AG)32	83.33	100	69.23
NW_020822526.1	17,494,091	(AC)16	17.65	57.14	0
NW_020822526.1	4,205,760	(ATGT)11	16.67	37.5	0
NW_020822529.1	28,253,360	(TG)23	91.67	100	86.67
NW_020822529.1	16,865,510	(TC)27	42.86	54.55	0
NW_020822530.1	10,618,047	(TG)22	18.18	28.57	0
NW_020822531.1	6,287,546	(AC)27	90.91	93.75	81.82
NW_020822531.1	1,037,419	(GT)44	45.45	50	12.5
NW_020822544.1	4,160,116	(AAC)5	22.73	60	0
NW_020822567.1	4,418,183	(TG)29	91.67	100	80
NW_020822567.1	6,942,408	(AC)24	25	35.29	0
NW_020822567.1	3,062,112	(CA)7	17.39	29.41	3.85
NW_020822567.1	1,362,729	(GA)24	15.38	25	0
NW_020822570.1	21,823,495	(TG)28	14.29	44.44	0
NW_020822591.1	7,123,790	(TATT)6	94.44	100	80
NW_020822591.1	7,780,615	(TAAA)8	33.33	37.5	0
NW_020822591.1	14,941	(GA)34	9.09	25	0
NW_020822591.1	8,820,175	(TTAT)11	9.52	27.27	0
NW_020822592.1	4,061,125	(AC)31	42.86	44.44	0
NW_020822595.1	5,415,636	(CAAA)6	37.93	38.46	7.69
NW_020822597.1	5,771,854	(TG)31	88.24	94.44	75
NW_020822601.1	72,176,322	(CA)8	91.18	100	85.71
NW_020822601.1	61,385,941	(AC)19	21.43	22.73	0
NW_020822602.1	6,751,043	(TC)30	91.67	96.55	83.33
NW_020822602.1	9,765,422	(AATA)7	15.38	15.79	0
NW_020822603.1	9,112,470	(AC)25	92.31	100	66.67
NW_020822604.1	8,845,685	(GT)6	10	37.5	0
NW_020822610.1	821,820	(AC)27	31.25	41.18	10
NW_020822614.1	8,982,333	(ATAG)16	13.64	13.79	0
NW_020822629.1	5,127,703	(AG)28	12.5	20	0
NW_020822629.1	6,170,860	(CA)22	12.5	33.33	0
NW_020822634.1	2,791,562	(TC)33	95.24	100	62.5
NW_020822638.1	3,030,009	(AC)21	92.31	100	58.33
NW_020822698.1	6,755,673	(GT)27	90	100	80
NW_020822698.1	121,373	(TG)30	28.57	37.5	0
NW_020822785.1	9557	(CA)14	14.29	75	0
NW_020822967.1	23,702	(CA)17	15.38	33.33	9.09
NW_020823044.1	10,660	(TTG)6	15.29	15.45	2.53
NW_020823531.1	97,528	(AG)22	11.76	15.22	5.41
NW_020823768.1	45,602	(TC)26	93.75	95.83	78.95
NW_020824031.1	35,819	(AC)23	20	25	0
NW_020824065.1	30,045	(AG)6	12	14.29	4.65

## Discussion

Ammonia is a common metabolic waste product in cell cultures. The accumulation of ammonia most often leads to decreased cell and recombinant protein productivity. Typical fed-batch cultures last for 14 to 20 days, where in the exponential phase, cell division can occur daily. De novo mutations that occur early in culture will be amplified and have the potential to dominate the cell population as the culture approaches harvest. In this study, two ammonia stresses (10 mM) and (30 mM) were used to investigate the genotoxic effects of this byproduct on CHO cell fed-batch cultures. Further, the role of ammonia stress on genome instability was investigated. Despite a relatively short exposure duration of 72 h, MSI loci were identified, which have the potential to be biomarkers for genome instability.

### Metabolic response

The VCD, cell viability, and metabolic profiles indicated that the 30 mM ammonia stress significantly impacted the culture health, as the characteristic cell growth and metabolic profiles were significantly different from the control cultures. The effects of the 10 mM ammonia stress were less profound, yet the metabolic profiles and protein productivity were more sensitive to these changes than the cell viability and VCD profiles. At the time of sampling for genetic analysis (3.5 days), VCDs for the control and 10 mM cultures appeared to be matched, whereas the 30 mM ammonia-stressed cultures had lower VCDs. The decreased consumption of alanine observed for the ammonia-stressed cultures was the only metabolic difference observed at sampling for the whole genome sequencing. Alanine metabolic changes are known to occur under ammonia stress [39]. Therefore, the whole genome sequencing would identify changes due to the ammonia stresses, and not due to potential other culture condition differences that might accumulate.

### Genome instability

Until now, efforts to characterize ammonia stress effects on CHO cells have mainly focused on transcriptome, proteome, and product characteristic changes [14, 16, 17, 40–44]. In this study, the effects of ammonia stress were further characterized by examining variants within functional genes and microsatellites. Whole genome sequencing allowed for variant SNPs and indels to be identified. Moreover, greater than 2300 high or moderate-impact novel gene variants were identified from the ammonia-stressed cultures that may impact cellular functions of critical pathways. KEGG and GO enrichment analyses confirmed that many of the variant genes affected pathways could lead to suboptimal clone performance. Though thousands of variant genes were identified, this list was narrowed to focus on genes pertaining to pathways involved in genome stability (Figs. 3 and 4, Table 1).

Alterations in critical genes responsible for a wide variety of processes such as transcription regulation, cell cycle regulation, tumor suppression, and signaling pathways may lead to global genome instability. De novo genomic SNPs and indels accumulating is typically the result of replication errors which can result from a variety of mechanisms such as replication stalling [45], replication fork collapse [46, 47], double-strand breaks [48, 49], environmental stressors, transcription regulation errors, or other replication errors [50]. All of these replication mechanisms can be linked to error correction fidelity of DNA repair mechanisms. These DNA repair errors, in turn, can lead to an accelerated variant accumulation rates and loss of genome stability [51]. Mutations, such as synonymous base changes in coding and regulatory regions, normally have little to no effect on gene transcription and translation, however, non-synonymous changes can have functional effects on the subsequent amino acid sequence and folding or function that ultimately can be linked to loss in cell viability.

Through text mining approaches, 273 of the variant genes found in the CHO genome were linked to human orthologs; whose function are related to genome stability maintenance. One gene identified is exceptionally well-known for its role in double-strand break repair and tumor suppression, *Brca1* (Table 1); loss of *Brca1* function has been associated with increased breast cancer incidence and metastasis, which demonstrates its critical function in maintaining stability [52]. Genome instability can be further exacerbated by the loss of tumor suppressor function. For example, *Lin9* (Table 1) is a tumor suppressor that inhibits DNA synthesis and acts synergistically with the well-known*Rb1* gene to prevent rapid, uncontrolled cell division [53]. Therefore, loss of function in *Lin9* can lead to cancer-like growth of mutant cells that would eventually dominate the culture population.

Some variant genes belonged to three significantly enriched KEGG pathways related to genome instability in humans - cellular senescence, cell cycle, and homologous recombination (Fig. 2). Cellular senescence occurs as a result of multiple stimuli such as DNA damage and oxidative stress [54]. By forcing the cells into a non-replicative state, senescence can severely limit the productivity of cell culture, especially when it occurs before or in the early exponential growth phase. The 30 mM stressed cultures had more genes enriched in the senescence pathway (Supplemental Table S6), which makes variant genes in this pathway a likely contributor to the poor growth observed. The second pathway, cell cycle, was observed to have significant enrichment in union genes of the 10 mM and 30 mM ammonia-stressed cultures. The cell cycle contains multiple checkpoints to ensure daughter cells are healthy and contain undamaged DNA [55, 56]. Significant enrichment in this pathway indicates that damaged or otherwise improperly replicated DNA could be passed on to daughter cells. Finally, the homologous recombination pathway repairs damage caused by double strand breaks by using an identical sequence as a template [57]. This repair method is much more accurate than non-homologous end joining and is less prone to variant generation [58].

It should be noted that while the mismatch repair (MMR) pathway was not found to be significantly enriched, three notable MMR genes accumulated variants: *Mlh3* (a *MutL* homolog), *Rpa1*, and *Abl1* (Supplemental Table S5). An impaired or inefficient MMR system can lead to the accumulation of mutations in functional genes over cell divisions that are critical to the cell’s survival and can lead to loss of genetic stability [59] or disease states, such as cancer [60]. The need for a highly conserved MMR system can be observed by the presence of multiple orthologs of *MutS* and *MutL* in eukaryotic genomes [61]. *MutS* binds to base mismatches or small indels [62, 63] while *MutL* is responsible for communicating the identification of mismatch events to downstream elements of MMR such as exonucleases [64].

### Microsatellite instability

Variants in genes that regulate the MMR pathway may be an origin to the cascade of events that leads to genome instability. When the MMR pathway in a cell is compromised, mistakes can occur and propagate indiscriminately across the genome as cell division occurs [61–63]. Unfaithful replication of genomic repeats, such as microsatellite repeats, have been used as effective biomarkers in predicting certain diseases, such as cancer In this study, we found 1022 microsatellites with variable repeat lengths in the WGS reads from ammonia stressed samples. We developed criteria and an approach to identify microsatellite loci that have variant length that can be attributed to ammonia stress. For each site, we considered read depth, mutation type, and frequency in affected and control samples to subset 124 candidate MSI loci that contain indels with a dose dependent response to the ammonia concentrations that were not observed in the control cultures. This set of 124 MSI loci represent a potential biomarker set that could have utility to predict genome instability in CHO cell cultures under stressful culture conditions.

## Conclusion

The accumulation of metabolic wastes, such as ammonia, can have a profound effect on CHO cell culture viability, transcriptome, and recombinant protein productivity. Additionally, past work, as well as this study, have observed shifts in growth patterns and metabolic profiles due to the ammonia stress. Further, in this study, it was observed that high levels of exogenous ammonia caused de novo mutations, such as SNPs and indels, within functional genes. More importantly, these mutations persisted throughout the culture population. Variants were identified in the genes that regulate critical cellular processes, such as DNA repair; which is a hallmark of genome instability. In addition to characterizing the microsatellite content of the Chinese hamster genome, potential MSI loci that exhibited unfaithful replication in the presence of exogenous ammonia were identified; these microsatellites could be utilized as a tool to diagnose genome instability in future work.

## Supplementary Information

**Additional file 1: Supplemental Table S1.** Complete list of SNP variants identified in each treatment group. **Supplemental Table S2.** Complete list of indel variants identified in each treatment group and initial mutation score calculations. **Supplemental Table S3.** Variant reads identified within functional genes. **Supplemental Table S4.** Variants identified within protein coding genes. **Supplemental Table S5.**Human-Chinese hamster gene orthologs that can be linked to genome instability via text mining.

**Additional file 2: Supplemental Table S6.** KEGG enrichment of variant genes that can be linked to genome instability. **Supplemental Table S7.** Statistically significant Biological Process GO terms of variant genes. **Supplemental Table S8.** Statistically significant Molecular Function GO terms of variant genes. **Supplemental Table S9.** Statistically significant Cellular Component GO terms of variant genes. **Supplemental Table S10.** Complete list of microsatelites found in the Chinese hamster genome. **Supplemental Table S11.** Microsatellites containing insertion and or deletion mutations. **Supplemental Table S12.** Indel loci with higher mutation frequencies in 30 mM ammonia-stressed cultures. **Supplemental Table S13.** Indel loci from table S12 where mutation frequency is higher in the 10 mM samples compared to the control. **Supplemental Table S14.** The intersection of dose-dependent indels (Table S13) and genome-wide microsatellites (S10). **Supplemental Table S15.** The 124 candidate microsatelite loci that exhibited dose-dependent variation in response to ammonia stress.

## Data Availability

The genomic sequence data generated and/or analyzed during the current study are available in the NCBI short read archive under BioProject: PRJNA579347 Submission ID: SAMN13108689. All other data generated or analyzed during this study are included in this published article and its supplementary information files.
